# Supplementary material for: Determining clinically relevant features in cytometry data using persistent homology
Source: PLoS Comput Biol. 2022 Mar 21;18(3):e1009931. doi: 10.1371/journal.pcbi.1009931 (PMC9009779; doi:10.1371/journal.pcbi.1009931)
Supplement: S1 Appendix — (PDF) [file pcbi.1009931.s001.pdf]

S1 Some omitted details

We have already introduced the concept of persistent homology. Its complete exposition is beyond the scope of this paper. We provide another intuitive example to illustrate its application in capturing prominent features hidden in a PCD. We refer to [1, 2] for a detailed exposition of topological persistence.

Suppose a set of points P is sampled along a curve with two 'holes'; see Fig. S1. If we grow balls of radius ϵ starting from zero around the sampled points we see that different holes get filled up at different times. The bigger hole gets filled at a much later time than the small one and the spurious ones. Persistent homology formalizes this idea of tracking the lifetime of topological features (homology groups).

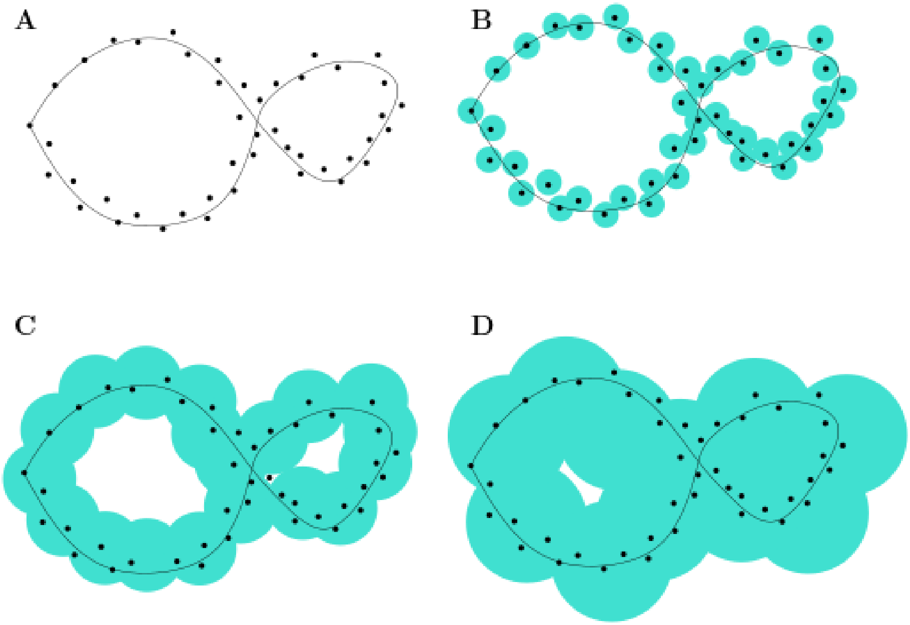

Figure S1. Intuition of persistence. (A) A set of points P sampled from a curve. **(B)** An Euclidean ball of radius ϵ is grown around each point in P. **(C)** As ϵ increases the smaller hole gets filled up. **(D)** The larger hole still 'persists' even though the smaller hole gets filled. Figures are adopted from [2, Fig. 4.2].

Definition S1.1 (Simplex)**.** A k-simplex σ is the convex hull of $k+1$ affinely independent point set P. We call k to be the dimension of σ and denote $dim(\sigma) = k$. A *face* σ' of σ is the convex hull of non-empty subset of P and this relation is given by $\sigma' \subseteq \sigma$.

In particular 0-simplex is a point, 1-simplex is an edge, 2-simplex is a triangle and so on.

Definition S1.2 (Simplicial Complex)**.** A set of simplices is defined as a simplicial complex $\mathcal{K}$ if the following two restrictions hold

- If $\sigma \in \mathcal{K}$ and $\sigma' \subseteq \sigma$ then $\sigma' \in \mathcal{K}$.

- For any two simplices $\sigma, \tau \in \mathcal{K}$, $\sigma \cap \tau$ is either empty or a face of both σ and τ.

The *dimension* of a simplicial complex $\mathcal{K}$ is the maximum dimension of any of its simplices.

Definition S1.3 (Filtration)**.** Given a Simplicial Complex $\mathcal{K}$ and a *monotonic* function $f : \mathcal{K} \to \mathbb{R}$ we get a nested sequence of subcomplex by denoting $\mathcal{K}_i = f^{-1}(-\infty, a_i]$ which is defined as *filtration*:

$$\phi = \mathcal{K}_0 \subseteq \mathcal{K}_1 \subseteq \mathcal{K}_2 \subseteq \ldots \subseteq \mathcal{K}_n = \mathcal{K}$$

By *monotonic* we mean that if $\sigma' \subseteq \sigma$, $f(\sigma') \leq f(\sigma)$.

References

1. Edelsbrunner H, Harer J. Computational topology: an introduction. American Mathematical Soc.; 2010.

2. Dey TK, Wang Y. Computational Topology for Data Analysis. Cambridge University Press; 2022. Available from: `https://books.google.com/books?id=PWtYEAAAQBAJ`.
