# Supplementary material for: Determining clinically relevant features in cytometry data using persistent homology
Source: PLoS Comput Biol. 2022 Mar 21;18(3):e1009931. doi: 10.1371/journal.pcbi.1009931 (PMC9009779; doi:10.1371/journal.pcbi.1009931)
Supplement: S2 Appendix — (PDF) [file pcbi.1009931.s002.pdf]

S2 Algorithms

Algorithm A DisPers

Input: c: Cytometry dataset, k: Nearest neighbors to consider, n: Number of samples
Output: $Dgm(c')$: Persistence diagram of c' sampled from cytometry data c

begin
    Subsample c
    $c' \leftarrow n$ samples from c
    $G \leftarrow$ GEN-COMPLETE-GRAPH(c', k)
    $Dgm(c') \leftarrow$ COMPUTE-PERSISTENCE(G)
    return $Dgm(c')$
end

Since a graph is a 1-dimensional simplicial complex, for a graph with m edges and n vertices, we can compute its 0-dim persistence in $\mathcal{O}(m \log n)$ time with Kruskal like minimum spanning tree algorithm [1].

Algorithm B Gen-Complete-Graph

Input: c: Sampled cytometry data, k: Nearest neighbors to consider
Output: $G(V, E)$: Complete Weighted Graph on c

procedure GEN-COMPLETE-GRAPH
    $G(V, E) \leftarrow \phi$ ▷ V is the set of nodes and E is the set of edges of G
    for all $v \in c$ **do**
        Compute $v_1, v_2, \ldots, v_k$, k-nearest neighbors of v
        $w(v) \leftarrow -\frac{1}{k}\sqrt{\sum_i \|v - v_i\|^2}$ ▷ $w(v)$ denotes vertex weight
        $V(G) \leftarrow V(G) \bigcup \big(v, w(v)\big)$
    end for
    for all $\{u, v\} \in c \times c$ and $u \neq v$ **do**
        $e \leftarrow \{u, v\}$
        $w(e) \leftarrow \|u - v\|$ ▷ $w(e)$ denotes weight of the edge
        $E(G) \leftarrow E(G) \bigcup (e, w(e))$
    end for
    return $G(V, E)$
end procedure

Algorithm C shows the steps of Persistence computation. Consider a generic step when an edge $e \in G$ is introduced in the minimum spanning forest that joins two forests rooted at nodes v_0 and v_1. For the new tree we will choose the root as node having smaller weight and the edge e pairs with the node having larger weight. Essentially we are choosing to kill the youngest connected component created by, with a little abuse of notation, the vertex $argmax(w(v_0), w(v_1))$. We define persistence of the edge as

$$p(e) = w(e) - \max\{w(v_0), w(v_1)\} \tag{1}$$

It is important to point out that an edge may or may not necessarily kill a connected component. If it does not kill a connected component it definitely creates a 1-cycle and we pair the edge with a special vertex with $w(v) = \infty$ and define $pers(e) = \infty$.

Algorithm C Compute Persistence Diagram

Input: $G(V, E)$: Complete weighted graph on sampled cytometry data c
Output: $Dgm(c)$: Persistence Diagram

procedure COMPUTE-PERSISTENCE
  $E' \leftarrow$ Sort E in increasing order of $w(e)$ with $e \in E$
  $P_0 \leftarrow \phi$ ▷ P_0 tracks 0-dim birth and death
  $P_1 \leftarrow \phi$ ▷ P_1 tracks 1-dim birth
  for all $e = (u, v) \in E'$ **do**
    $Root_0 \leftarrow find(u)$
    $Root_1 \leftarrow find(v)$
    if $Root_0 \neq Root_1$ **then**
      $birth \leftarrow max\{w(Root_0), w(Root_1)\}$ ▷ e kills youngest homology class
      $death \leftarrow w(e)$
      $pers(e) \leftarrow death - birth$
      $merge(Root_0, Root_1)$
      $P_0 \leftarrow P_0 \cup (birth, death)$
    else
      $P_1 \leftarrow P_1 \cup (w(e), \infty)$ ▷ e is a creator and creates a 1-cycle
    end if
  end for
  $Dgm(c) \leftarrow \{P_0, P_1\}$
  return $Dgm(c)$
end procedure

References

1. Edelsbrunner H, Harer J. Computational topology: an introduction. American Mathematical Soc.; 2010.
