# Supplementary figures and images for: Determining clinically relevant features in cytometry data using persistent homology
Source: PLoS Comput Biol. 2022 Mar 21;18(3):e1009931. doi: 10.1371/journal.pcbi.1009931 (PMC9009779; doi:10.1371/journal.pcbi.1009931)

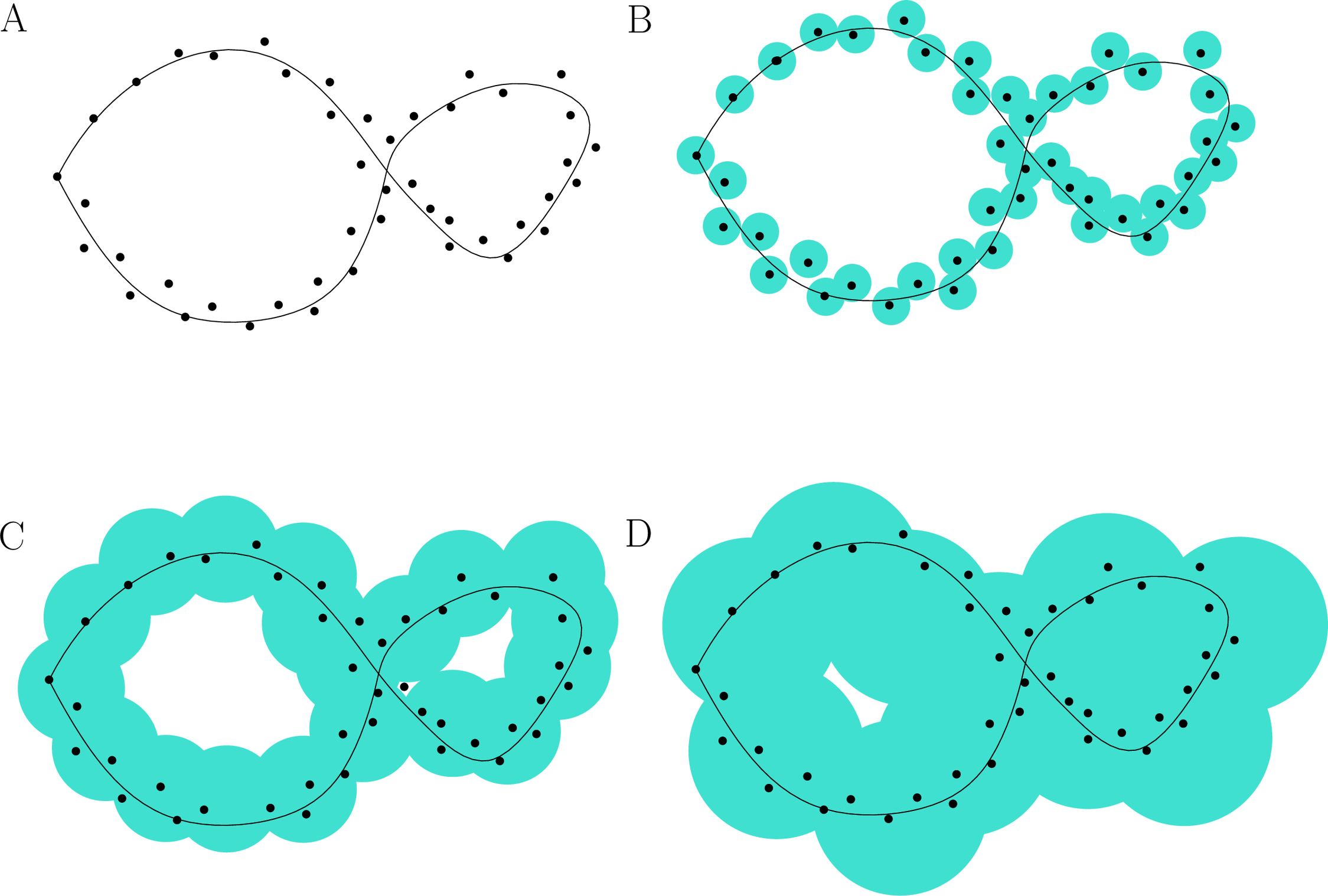

Supplement: S1 Fig — (A) A set of points P sampled from a curve. (B) An Euclidean ball of radius ϵ is grown around each point in P. (C) As ϵ increases the smaller hole gets filled up. (D) The larger hole still ‘persists’ even though the smaller hole gets filled. Figures are adopted from [26, Fig 4.2]. (TIF) [file pcbi.1009931.s003.tif]

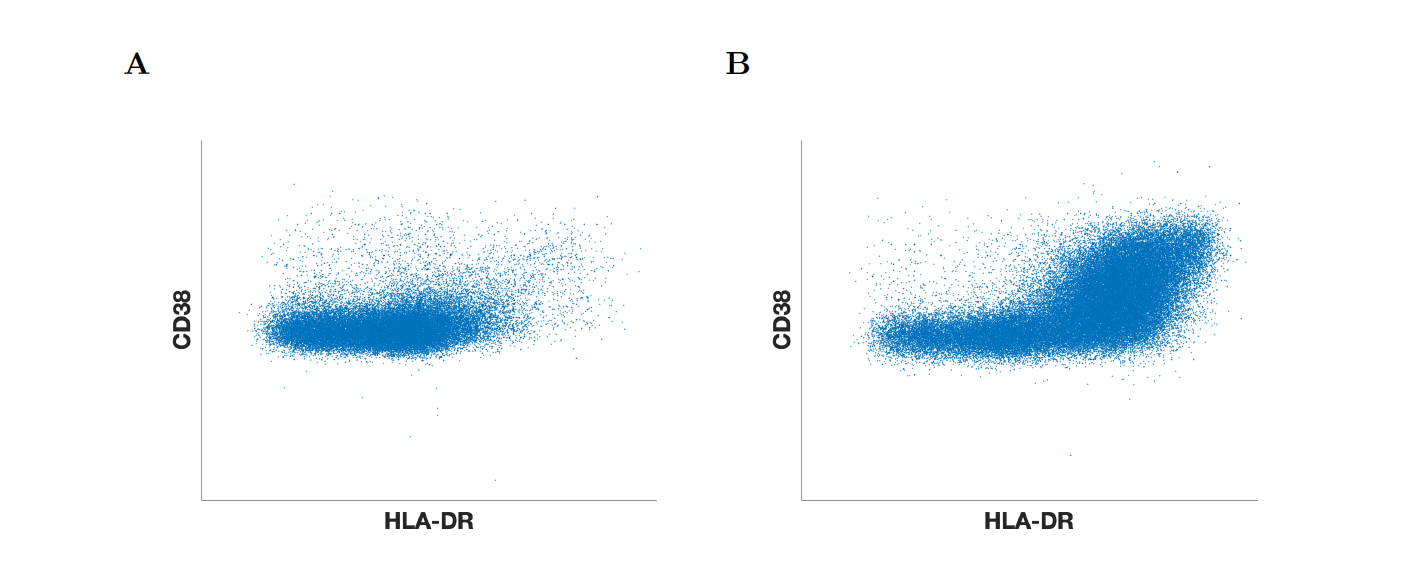

Supplement: S2 Fig — Transformed scatter plot for HLA-DR/CD38 axes for CD8+ T cell PCD in a singular (A) healthy donor and (B) COVID-19 patient. This plot demonstrates the ‘elbow’ found by the authors in [3]. The x-axis is asinh(HLA-DR/200) and the y-axis is asinh(CD38/500). (TIF) [file pcbi.1009931.s004.tif]

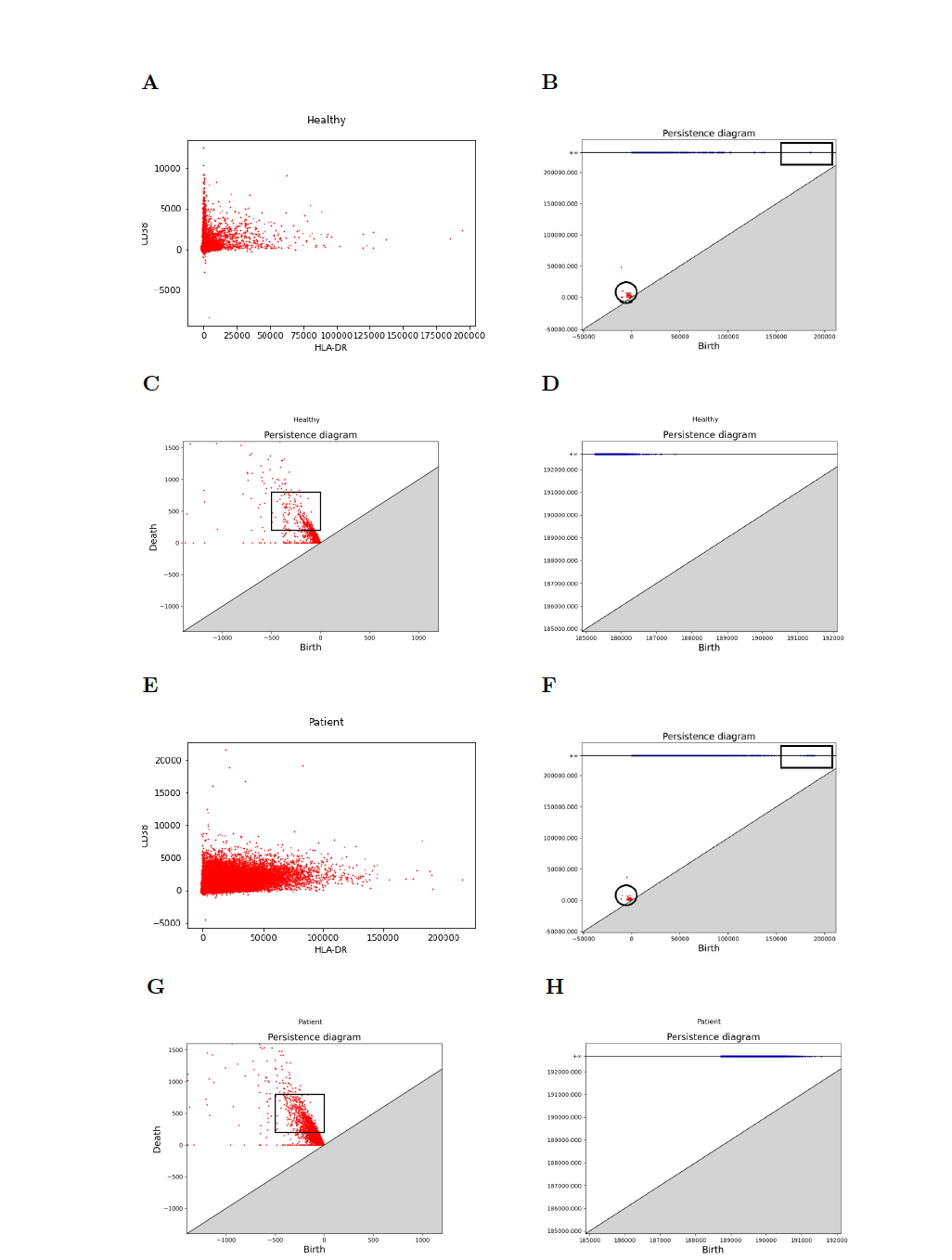

Supplement: S3 Fig — (A) Point cloud for individual healthy control in HLA-DR/CD38 expression levels. (B) Complete persistence diagram for the healthy control shown in (A). Boxes indicate zoomed regions for figures (C) and (D); (C) Zoomed in region from (B) of H0 persistence diagram. Box shows area of low density compared to patient persistence diagram; (D) Zoomed in region from (B) of H1 persistence diagram; (E)-(H) Same as (A)-(D), but for an individual COVID-19 patient. The box in (G) is more densely populated than the identical box in (C). (TIF) [file pcbi.1009931.s005.tif]

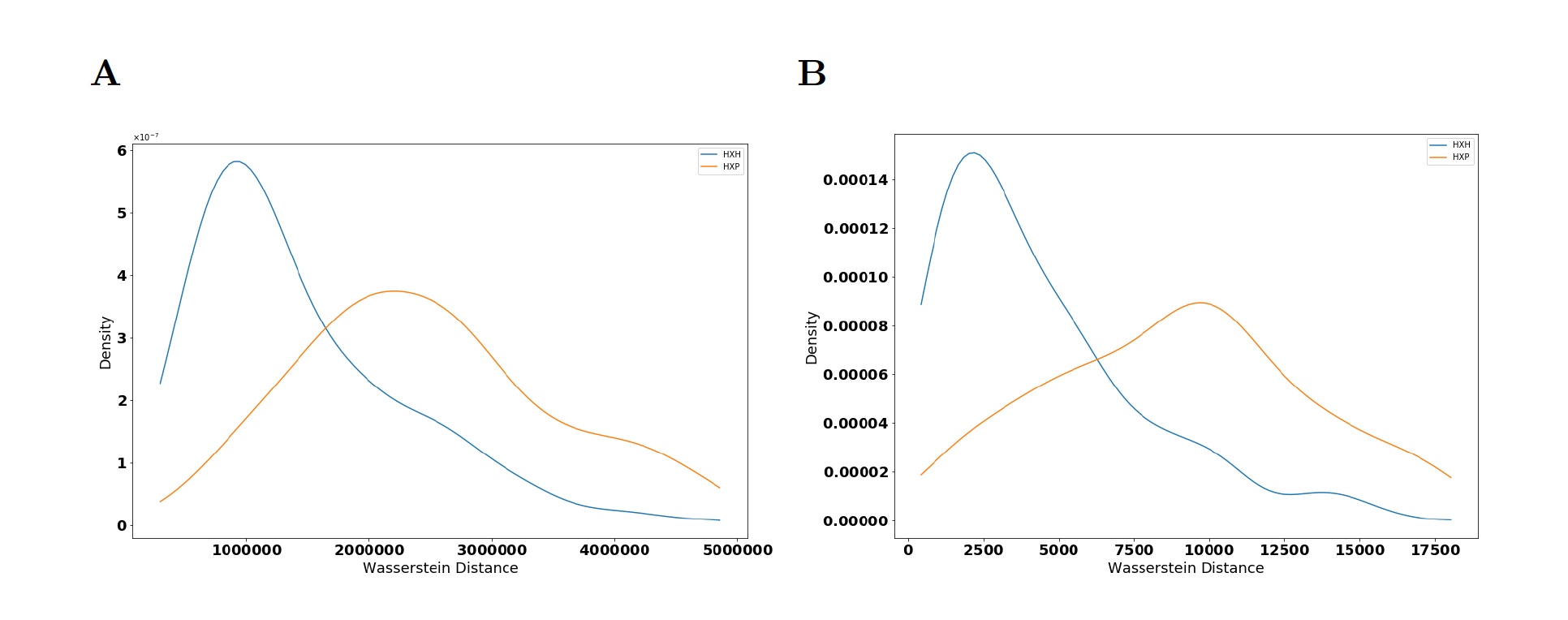

Supplement: S4 Fig — Distributions of Wasserstein distances between (A) H0-persistence diagrams (p = 6.75 × 10^−20^, QFD = 0.190) and (B) H1-persistence diagrams (p = 4.74 × 10^−24^, QFD = 0.220) for CD8+ T cells. Distances between pairs of healthy controls (H × H) and pairs of a healthy control and a COVID-19 patient (H × P) are overlaid. Persistence diagrams are calculated from point clouds in the T-bet, Eomes, and Ki-67 axes. This figure plots distributions of 200 randomly selected pairs, while Fig 5 plots distributions of 100 randomly selected pairs. (TIF) [file pcbi.1009931.s006.tif]

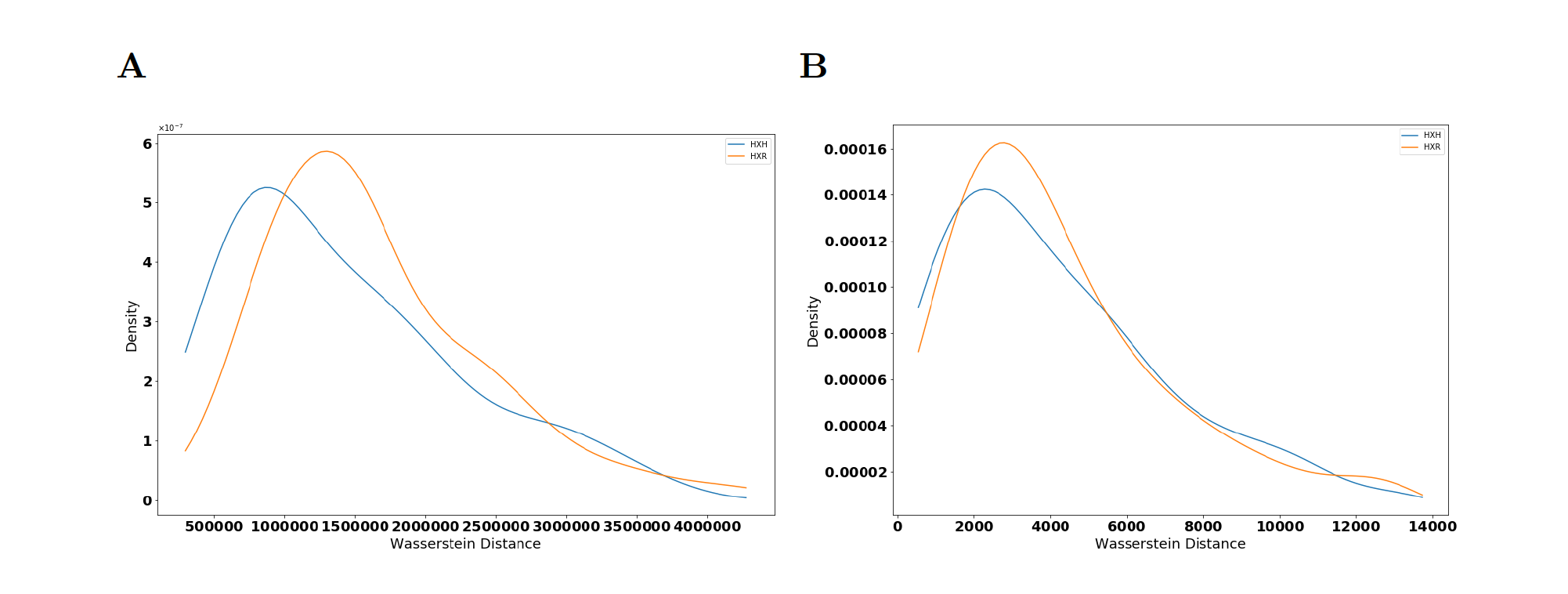

Supplement: S5 Fig — Distributions of Wasserstein distances between (A) H0-persistence diagrams (p = 0.131, QFD = 0.005) and (B) H1-persistence diagrams (p = 0.344, QFD = 0.001) for CD8+ T cells. Distances between pairs of healthy controls (H × H) and pairs of a healthy control and a individual that recovered from COVID-19 (H × R) are overlaid. Persistence diagrams are calculated from point clouds in the T-bet, Eomes and Ki-67 axes. p-values are calculated from a 2-sided KS test. (TIF) [file pcbi.1009931.s007.tif]

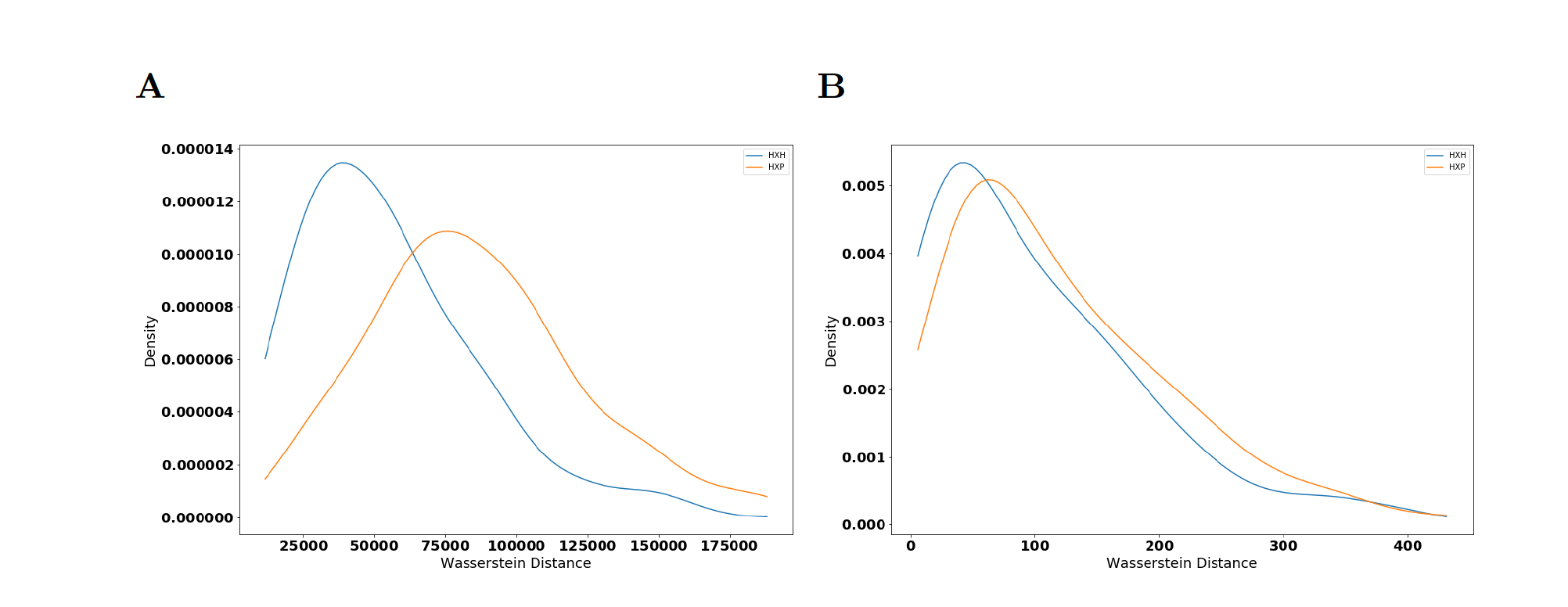

Supplement: S6 Fig — Distributions of Wasserstein distances between (A) H0-persistence diagrams (p = 2.75 × 10^−8^, QFD = 0.051) and (B) H1-persistence diagrams (p = 0.111, QFD = 0.022) for CD8+ T cells. Distances between pairs of healthy controls (H × H) and pairs of a healthy control and a COVID-19 patient (H × P) are overlaid. Persistence diagrams are calculated from point clouds in the IgD, CD4 and CD20 axes. p-values are calculated from a 2-sided KS test. (TIF) [file pcbi.1009931.s008.tif]

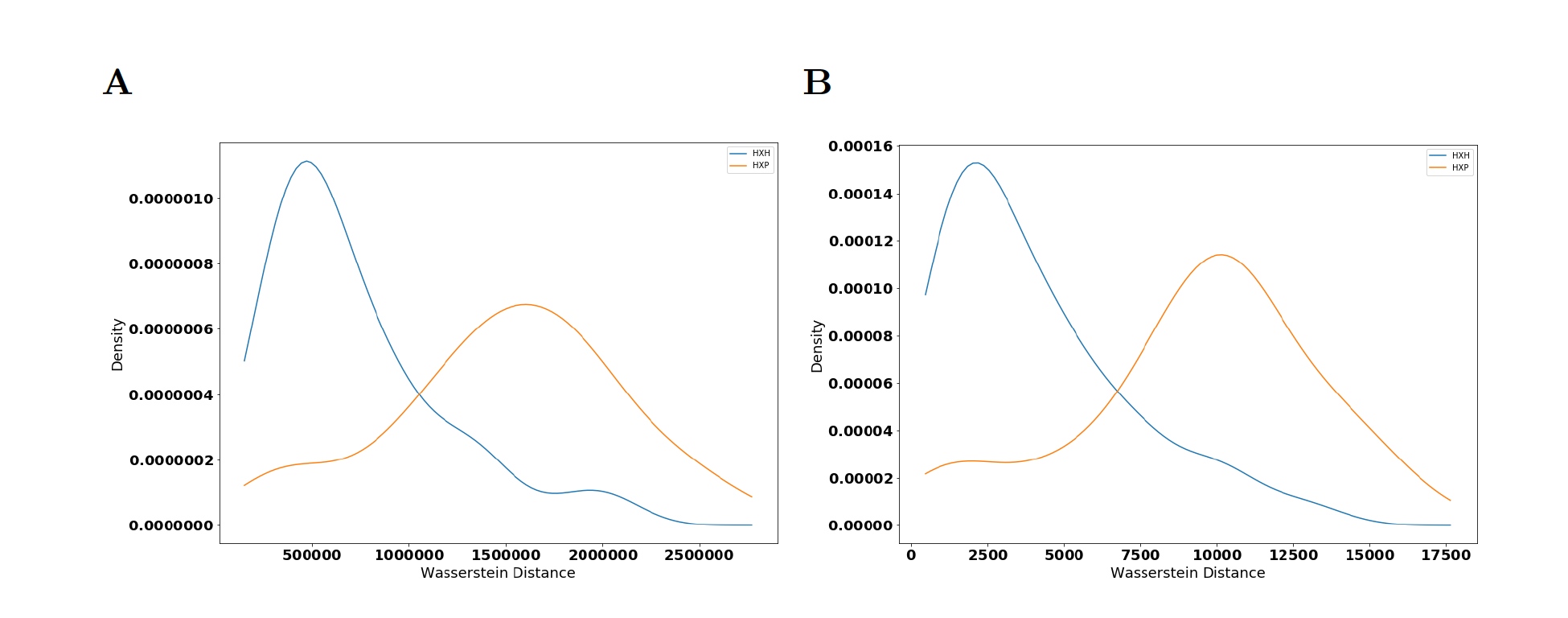

Supplement: S7 Fig — Distributions of Wasserstein distances between (A) H0-persistence diagrams (p = 6.31 × 10^−19^, QFD = 0.261) and (B) H1-persistence diagrams (p = 6.31 × 10^−19^, QFD = 0.276) for CD8+ T cells. Distances between pairs of healthy controls (H × H) and pairs of a healthy control and a COVID-19 patient (H × P) are overlaid. Persistence diagrams are calculated from point clouds in the T-bet and Eomes axes. p-values are calculated from a 2-sided KS test. (TIF) [file pcbi.1009931.s009.tif]

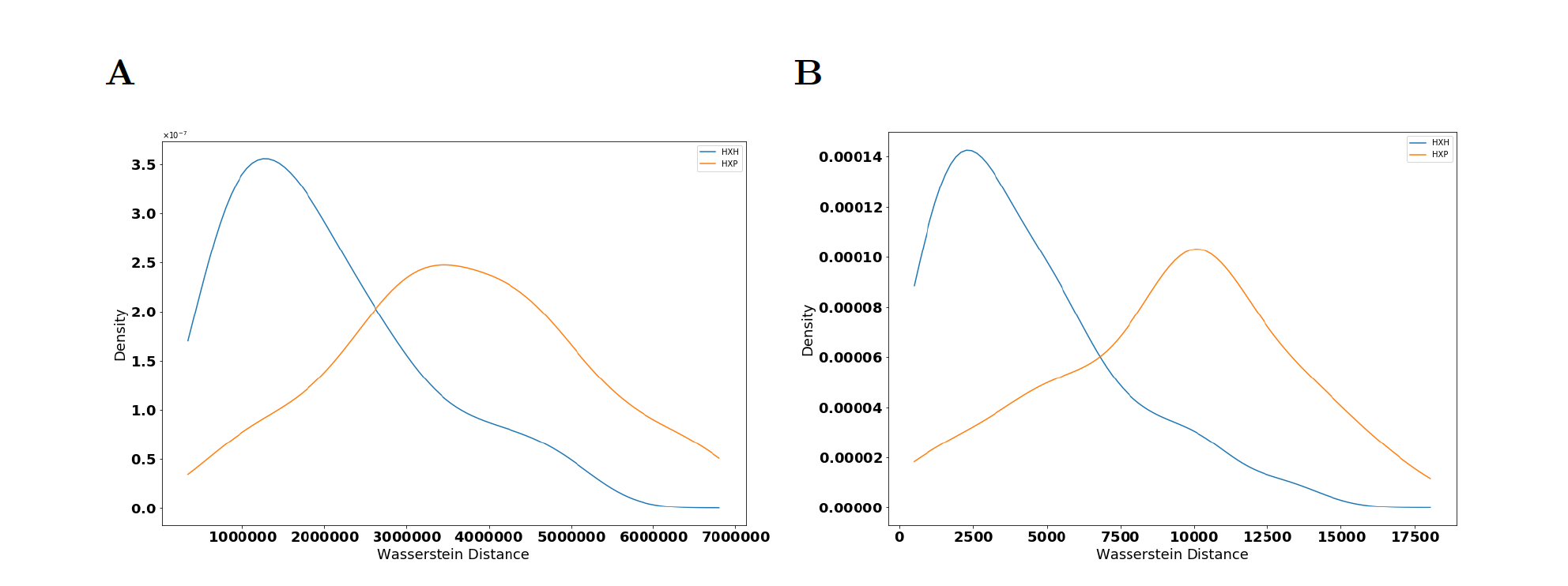

Supplement: S8 Fig — Distributions of Wasserstein distances between (A) H0-persistence diagrams (p = 3.35 × 10^−13^, QFD = 0.267) and (B) H1-persistence diagrams (p = 3.04 × 10^−14^, QFD = 0.265) for CD8+ T cells. Distances between pairs of healthy controls (H × H) and pairs of a healthy control and a COVID-19 patient (H × P) are overlaid. Persistence diagrams are calculated from point clouds in the T-bet, Eomes, Tox and TCF-1 axes. p-values are calculated from a 2-sided KS test. (TIF) [file pcbi.1009931.s010.tif]

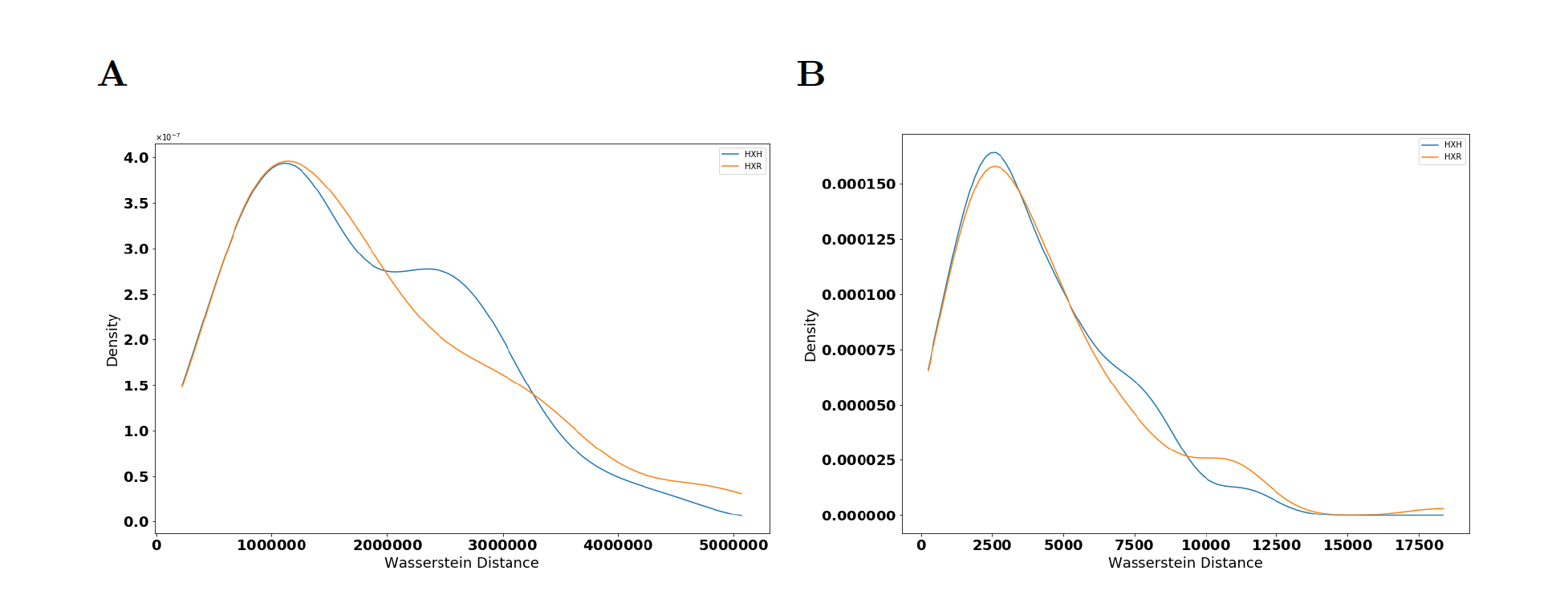

Supplement: S9 Fig — Distributions of Wasserstein distances between (A) H0-persistence diagrams (p = 0.908, QFD = 0.002) and (B) H1-persistence diagrams (p = 0.994, QFD = 0.001) for CD8+ T cell. Distances between pairs of healthy controls (H × H) and pairs of a healthy control and a individual that recovered from COVID-19 (H × R) are overlaid. Persistence diagrams are calculated from point clouds in the CD45RA, Eomes and TCF-1 axes. These 3 proteins are the best distinguishing features for the XGBoost classifier to distinguish cells from healthy controls from those from recovered individuals. p-values are calculated from a 2-sided KS test. (TIF) [file pcbi.1009931.s011.tif]

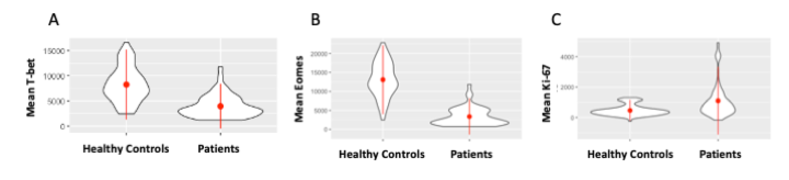

Supplement: S10 Fig — PDFs for (A) T-bet, (B) Eomes, and (C) Ki-67 for CD8+ T cell PCDs in each healthy control and COVID-19 patient shown using violin plots. The thickness of the “violin” denotes the value of the pdf. Data distributions are calculated from the mean protein abundances across all non-naïve CD8+ T cells for each individual. Red dots represent the mean of the data, and red lines represent the standard deviation. (TIF) [file pcbi.1009931.s012.tif]

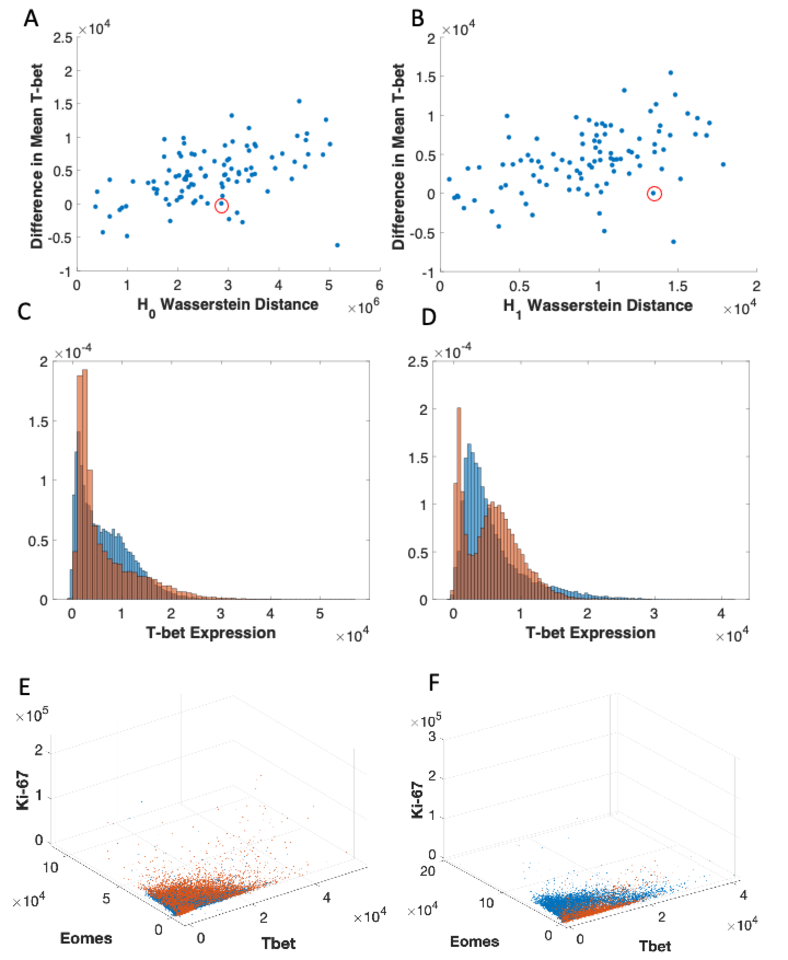

Supplement: S11 Fig — (A-B) Scatter plot showing the relationship between Wasserstein distance between H0 and H1 persistence diagrams and the difference in mean T-bet abundance for CD8+ T cells for random pairs of healthy controls and COVID-19 patients. Red circles highlight a pair of individuals that generated a large Wasserstein distance despite a small difference in mean T-bet expressions, which are further analyzed in (C-F). (C-D) Histograms showing the T-bet expression of non-naïve CD8+ T cells for the healthy control (blue) and COVID-19 patient (red) from the points circled above in (A-B). (E-F) Scatter plots showing the T-bet, Eomes, and Ki-67 abundances for the healthy control (blue) and COVID-19 patient (red) from the points circled in (A-B). (TIF) [file pcbi.1009931.s013.tif]

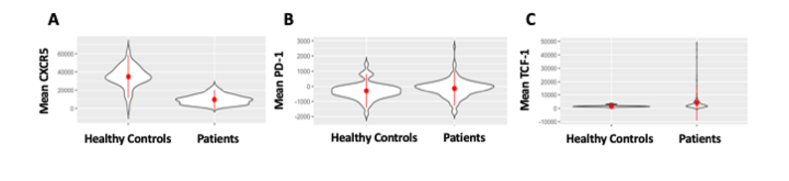

Supplement: S12 Fig — PDFs for (A) CXCR5, (B) PD-1, and (C) TCF-1 in B cells of each healthy control and COVID-19 patient shown using violin plots. The thickness of the “violin” denotes the value of the pdf. Data distributions are calculated from the mean protein abundances across all B cells for each individual. Red dots represent the mean of the data, and red lines represent the standard deviation. (TIF) [file pcbi.1009931.s014.tif]

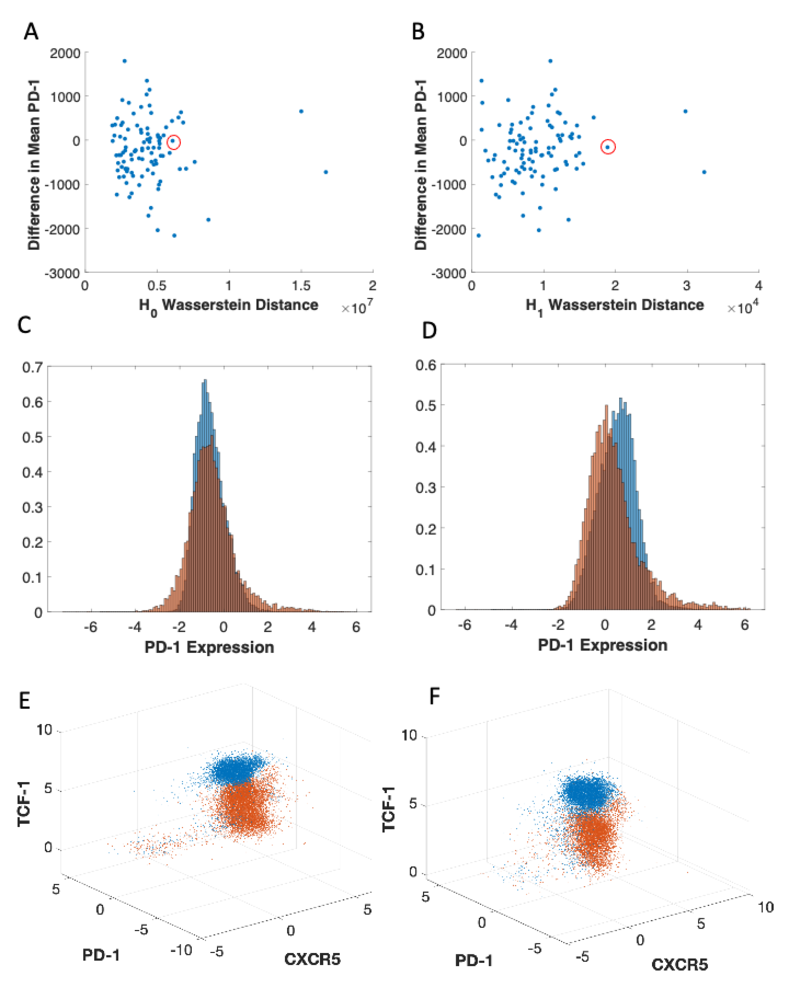

Supplement: S13 Fig — (A-B) Scatter plot showing the relationship between Wasserstein distance between H0 and H1 persistence diagrams and the difference in mean PD-1 abundance for B cells for random pairs of healthy controls and COVID-19 patients. Red circles highlight a pair of individuals that generated a large Wasserstein distance despite a small difference in mean PD-1 expressions, which are further analyzed in (C-F). (C-D) Histograms showing the PD-1 expression of non-naïve B cells for the healthy control (blue) and COVID-19 patient (red) from the points circled above in (A-B). (E-F) Scatter plots showing the CXCR5, PD-1, and TCF-1 abundances for the healthy control (blue) and COVID-19 patient (red) from the points circled in (A-B). Protein expression axes in (C)-(F) are scaled to asinh(x/150), where x is the expression of the given protein. (TIF) [file pcbi.1009931.s015.tif]

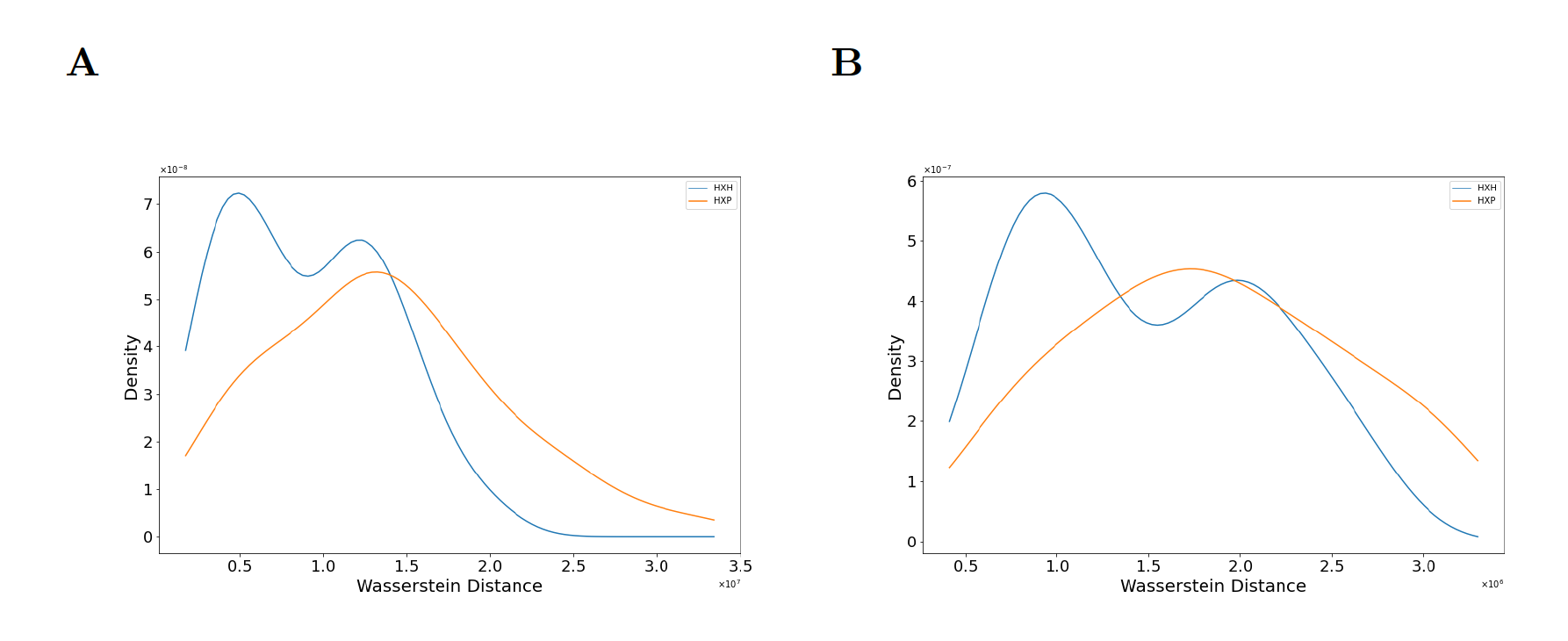

Supplement: S14 Fig — (A) Shows distributions of Wasserstein distance between H0-persistence diagrams for H×H (blue line) and H×P (orange line) pairs (p = 1.20 × 10^−4^, QFD = 0.0575) for CD8+ T cells. (B) Shows distributions of Wasserstein distance between H1-persistence diagrams for H×H (blue line) and H×P (orange line) for the same pairs in (A) (p = 3.73 × 10^−3^, QFD = 0.0343). (TIF) [file pcbi.1009931.s016.tif]

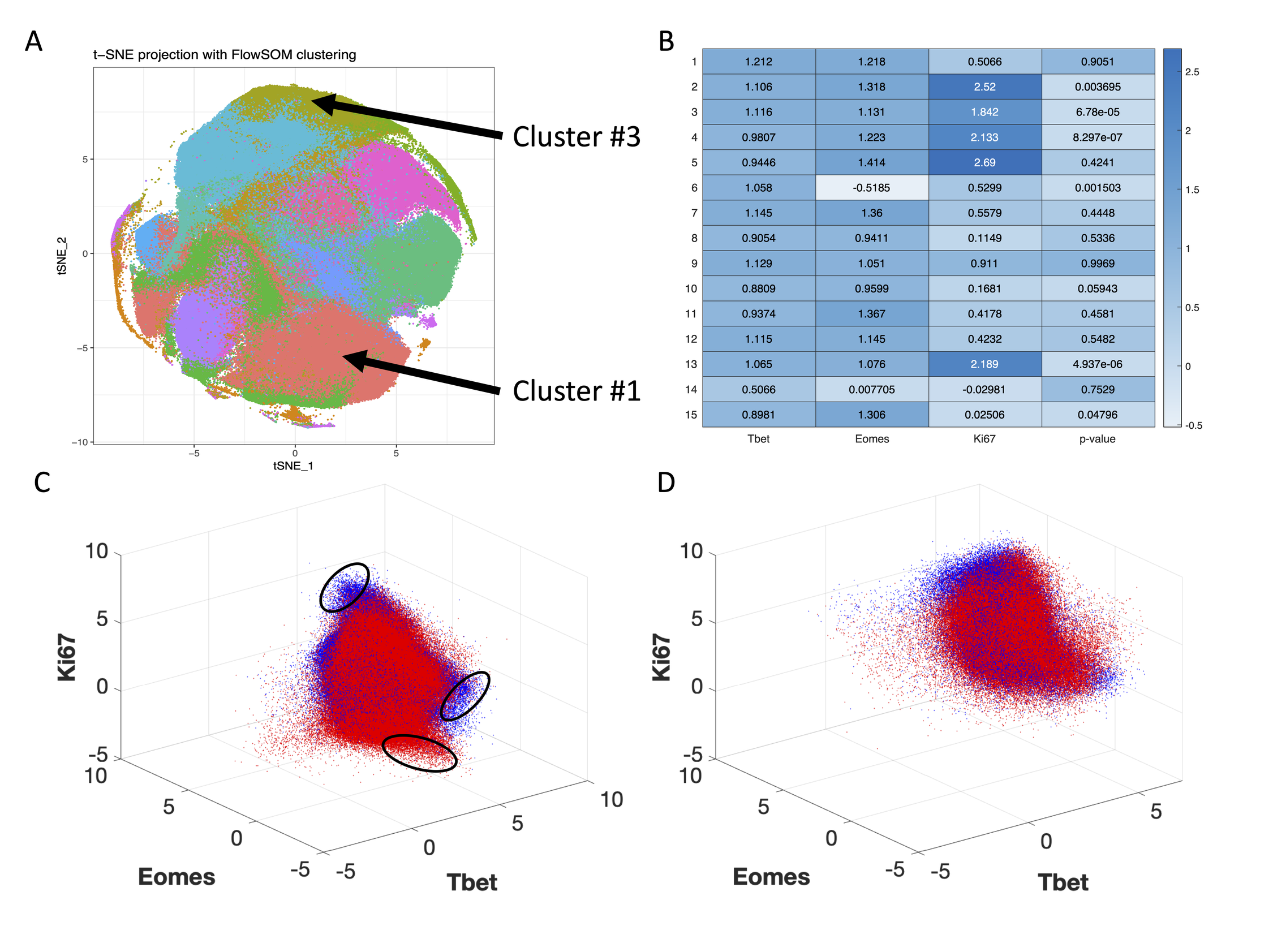

Supplement: S15 Fig — (A) t-SNE projection of protein expression data for CD8+ T cell PCD in Mathew et al. Each point represents a cell with 25 protein expressions. Colors represent 15 clusters identified by FlowSOM. Cluster #1 and Cluster #3 are selected for further topological analysis. (B) Heatmap showing scaled MFI for T-bet, Eomes, and Ki-67 for each cluster. Each entry in the first three columns is the MFI scaled by the average MFI of the column. The fourth column shows p-values determining differential expression of the cluster between healthy controls and COVID-19 patients. Note that Cluster #1 has p > 0.05 and Cluster #3 has p < 0.05. (C-D) Scatter plots showing the T-bet, Eomes, and Ki-67 abundances for all healthy controls (blue) and COVID-19 patients (red) from the cells in Cluster #1 (C) and Cluster #3 (D). Black circles in (C) indicate regions of single cell protein expressions which contribute to the differences in the PCD structure for the FlowSOM clusters. Axes are scaled to asinh(x/150), where x is the expression of the given protein. (TIF) [file pcbi.1009931.s017.tif]

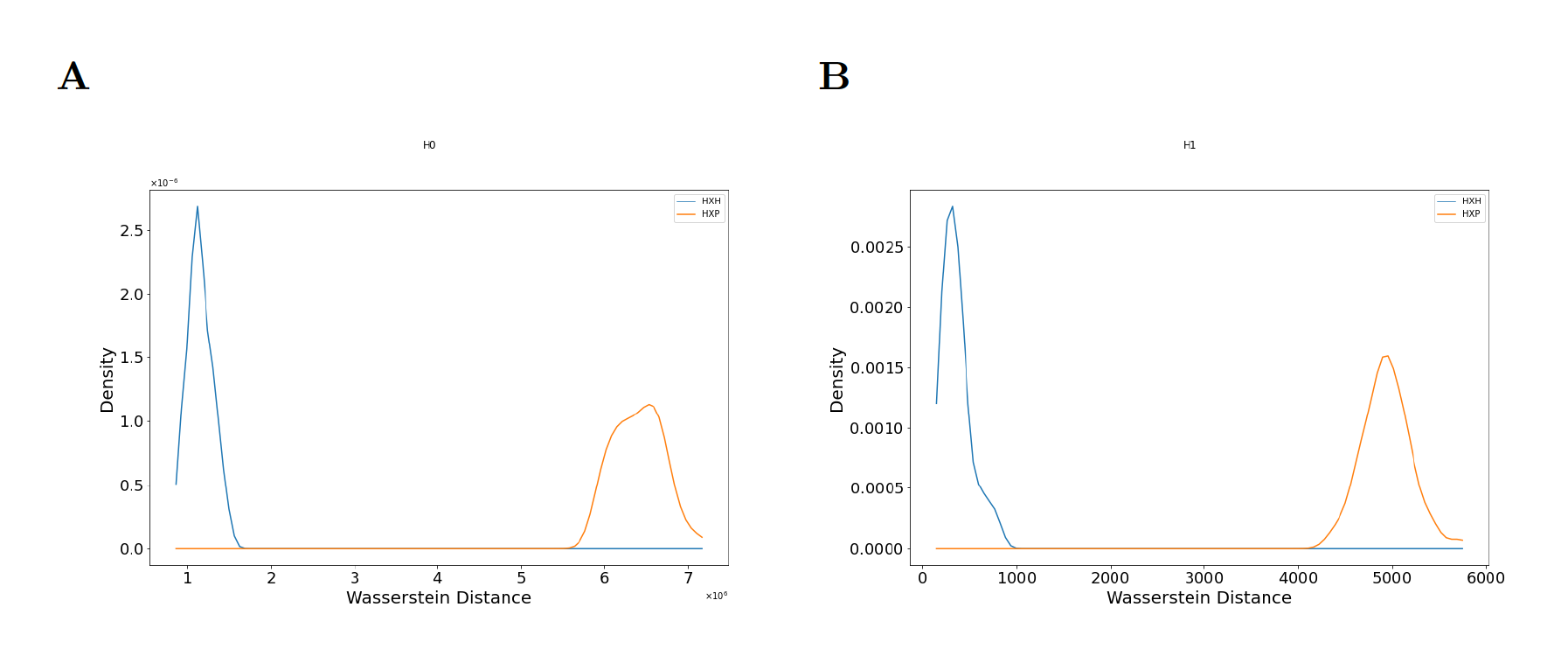

Supplement: S16 Fig — (A) Shows distributions of Wasserstein distance between H0-persistence diagrams for H×H (blue line) and H×P (orange line) pairs (p = 2.20 × 10^−59^, QFD = 1.604). (B) Shows distributions of Wasserstein distance between H1-persistence diagrams for H×H (blue line) and H×P (orange line) for the same pairs in (A) (p = 2.21 × 10^−59^, QFD = 1.573). (TIF) [file pcbi.1009931.s018.tif]

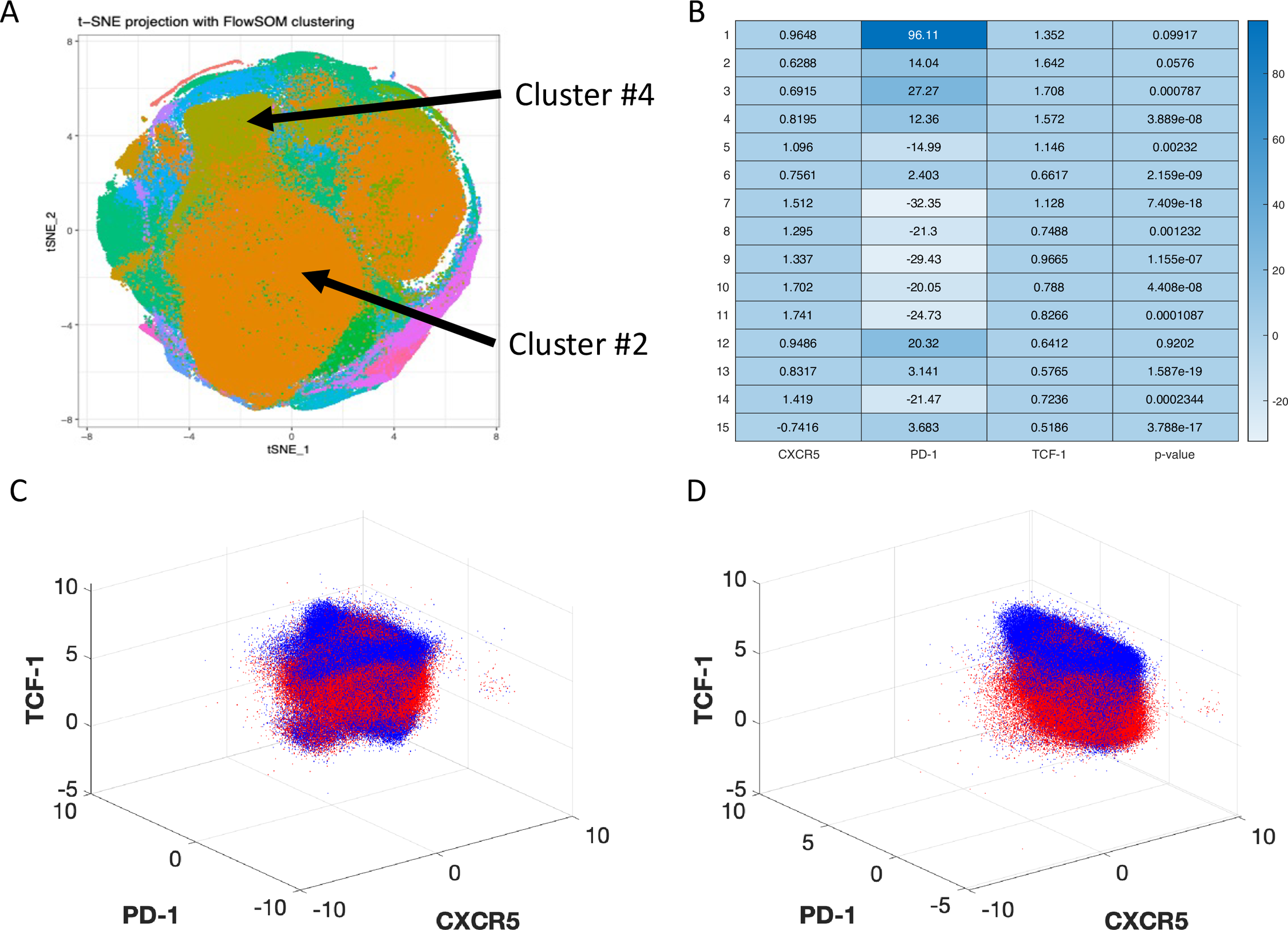

Supplement: S17 Fig — (A) t-SNE projection of protein expression data for B cell PCD in Mathew et al. Each point represents a cell with 25 protein expressions. Colors represent 15 clusters identified by FlowSOM. Cluster #2 and Cluster #4 are selected for further topological analysis. (B) Heatmap showing scaled MFI for CXCR5, PD-1, and TCF-1 for each cluster. Each entry in the first three columns is the MFI scaled by the average MFI of the column. The fourth column shows p-values determining differential expression of the cluster between healthy controls and COVID-19 patients. Note that Cluster #2 has p > 0.05 and Cluster #4 has p < 0.05. (C-D) Scatter plots showing the CXCR5, PD-1, and TCF-1 abundances for all healthy controls (blue) and COVID-19 patients (red) from the cells in Cluster #2 (C) and Cluster #4 (D). Axes are scaled to asinh(x/150), where x is the expression of the given protein. (TIF) [file pcbi.1009931.s019.tif]
